# Performance of ECG-Derived Digital Biomarker for Screening Coronary Occlusion in Resuscitated Out-of-Hospital Cardiac Arrest Patients: A Comparative Study between Artificial Intelligence and a Group of Experts

**DOI:** 10.3390/jcm13051354

**Published:** 2024-02-27

**Authors:** Min Ji Park, Yoo Jin Choi, Moonki Shim, Youngjin Cho, Jiesuck Park, Jina Choi, Joonghee Kim, Eunkyoung Lee, Seo-Yoon Kim

**Affiliations:** 1 Department of Emergency Medicine, Ajou University School of Medicine, Suwon-si 16499, Republic of Korea; parkmjem@gmail.com (M.J.P.); moonki.shim@gmail.com (M.S.); seoyoon426@aumc.ac.kr (S.-Y.K.); 2 Department of Cardiology, Seoul National University Bundang Hospital, Seongnam-si 13620, Republic of Korea; cho_y@snubh.org (Y.C.); cardio.jspark@gmail.com (J.P.); mdmiki0552@gmail.com (J.C.); 3 Department of Emergency Medicine, Seoul National University Bundang Hospital, Seongnam-si 13620, Republic of Korea; joonghee79@gmail.com (J.K.); heayang0418@naver.com (E.L.); 4 Big Data Center, Seoul National University Bundang Hospital, Seongnam-si 13620, Republic of Korea

**Keywords:** out-of-hospital cardiac arrest, ST elevation myocardial infarction, artificial intelligence, electrocardiography

## Abstract

Acute coronary syndrome is a significant part of cardiac etiology contributing to out-of-hospital cardiac arrest (OHCA), and immediate coronary angiography has been proposed to improve survival. This study evaluated the effectiveness of an AI algorithm in diagnosing near-total or total occlusion of coronary arteries in OHCA patients who regained spontaneous circulation. Conducted from 1 July 2019 to 30 June 2022 at a tertiary university hospital emergency department, it involved 82 OHCA patients, with 58 qualifying after exclusions. The AI used was the Quantitative ECG (QCG™) system, which provides a STEMI diagnostic score ranging from 0 to 100. The QCG score’s diagnostic performance was compared to assessments by two emergency physicians and three cardiologists. Among the patients, coronary occlusion was identified in 24. The QCG score showed a significant difference between occlusion and non-occlusion groups, with the former scoring higher. The QCG biomarker had an area under the curve (AUC) of 0.770, outperforming the expert group’s AUC of 0.676. It demonstrated 70.8% sensitivity and 79.4% specificity. These findings suggest that the AI-based ECG biomarker could predict coronary occlusion in resuscitated OHCA patients, and it was non-inferior to the consensus of the expert group.

## 1. Introduction

EMS-assisted out-of-hospital cardiac arrests (OHCAs) have an annual incidence of up to 35,000 cases in the United States, and the rate of survival-to-discharge is known to be approximately 9% [1]. Since cardiac causes are implicated as a major contributor to OHCA, representing around 60.8% [2], and as acute coronary syndrome (ACS) accounts for 16% of OHCA cases [3], previous studies have suggested that immediate coronary angiography (CAG) for the identification of individuals who require emergency coronary angioplasty could potentially improve patient survival by addressing reversible causes [4,5,6,7]. While the role of immediate CAG in patients with cardiac arrest without ST-segment elevation on electrocardiography (ECG) remains debatable [8,9,10], the importance of prompt revascularization for acute coronary occlusion in patients showing ST-segment elevation after return of spontaneous circulation (ROSC) remains unchanged [11,12].

An acute ST-elevation myocardial infarction (STEMI) is an event in which transmural myocardial ischemia results in myocardial injury or necrosis with typical changes in the ST segment on an ECG. This condition arises due to the occlusion of one or more coronary arteries, which are vital for supplying blood to the heart [13]. However, it is difficult to accurately identify such coronary occlusions from 12-lead ECGs in resuscitated OHCA patients. ECGs after ROSC can show various changes due to factors like electrolyte imbalances, post-arrest myocardial dysfunction, or intracranial bleeding, such as subarachnoid hemorrhages which can mimic or mask underlying coronary occlusion [14].

Recently, we have developed an artificial intelligence (AI) system (QCG™) that can extract various digital biomarkers from printed 12-lead ECGs. This biomarker demonstrated high accuracy in identifying acute total or near-total coronary occlusion in emergency department settings [15]. As the system has also been implemented on smartphones with camera functionality, it can be easily used in the resuscitation room. However, its accuracy for screening acute coronary occlusion in resuscitated OHCA patients has not been evaluated. Therefore, our aim was to assess the performance of the system’s occlusive myocardial infarction (MI) biomarker, qSTEMI, in screening for acute coronary occlusion in post-ROSC patients.

## 2. Materials and Methods

### 2.1. The AI Algorithm

The Quantitative ECG (QCG™) system (ARPI Inc., Seongnam-si, Gyenggi-do, Republic of Korea) version 1.0.1. comprises a heterogeneous set of deep learning-based ECG encoders designed to evaluate various cardiac conditions using printed ECGs [15]. We utilized its STEMI digital biomarker, qSTEMI, which is designed to assess acute coronary occlusion. This biomarker outputs a score between 0 and 100, which is correlated with the risk of coronary occlusion. The algorithm has been externally validated and has shown superior performance compared to a group of emergency physicians and cardiologists in detecting coronary occlusion [15]. 

### 2.2. Study Setting, Participants, and Data Preparation 

This retrospective observational study was conducted at a tertiary university hospital with an annual emergency department (ED) attendance of over 70,000 individuals. The study population included adult patients aged 18 years or older who presented to the ED with OHCA from 1 July 2019 to 30 June 2022. The inclusion criteria were patients who arrived at the emergency department with OHCA, underwent coronary angiography and underwent a 12-lead ECG within one hour post-ROSC. The exclusion criteria were as follows: (1) cases of ventricular tachycardia (VT), (2) pacemaker interference, and (3) cases where coronary angiography suggested vasospasm, where ST-elevations are indistinguishable from those of actual STEMI.

Medical records were reviewed to collect demographic details, pre-existing conditions, laboratory results, Utstein variables, initial post-ROSC ECGs, outcomes of CAG, and treatment outcomes, including the 30-day survival data. The laboratory results were obtained from the first blood test conducted immediately after ROSC and utilized to assess the patient’s initial state following ROSC. ECG evaluations utilized the first ECG conducted within one hour after ROSC, with all of these ECGs being performed prior to coronary angiography. The Institutional Review Board (IRB) of the hospitals approved the use of data.

### 2.3. Definitions

Cardiac arrest is defined as the cessation of cardiac mechanical activity and is confirmed by the absence of signs of circulation [16]. OHCA refers to cardiac arrest that occurs outside of a hospital setting. The presence of coronary occlusion was determined based on the results of coronary angiography performed after resuscitation. Near-total or total occlusion was defined as a positive case of acute coronary occlusion, while cases without such findings were defined as negative. However, if the report described the occlusion as chronic, it was considered an existing lesion and defined as a negative case.

### 2.4. Measurements of Human and AI Performance

Both experts and the AI system used the same ECG images for interpretation. These images were derived from screen captures of the waveform area of the original 12-lead ECG reports generated by MAC^®^ 5500 HD (General Electric, Boston, MA, USA) at a 25-mm paper speed and were accessed from the electronic medical records (EMR). To simulate a typical clinical scenario involving multiple experts in decision-making, a consensus score of a group of experts, including two board-certified emergency physicians and three board-certified cardiologists, was calculated. They were informed that the supplied ECGs were the initial ones obtained after ROSC in OHCA patients, without disclosure of any other details, such as age, gender, and underlying diseases. The participants reviewed the ECGs at their own pace and provided binary responses (“yes” or “no” for STEMI). The performance of the AI was assessed using the same ECGs (PNG format) as those used for human evaluation, obtaining qSTEMI. A QCG value of 50 or higher was considered a positive diagnosis for coronary occlusion.

### 2.5. Statistical Analysis 

Categorical variables were reported as frequencies and proportions. Continuous variables were reported as mean and standard deviation (SD) or median and interquartile range (IQR) as appropriate. Sensitivity, specificity, positive predictive value (PPV), and negative predictive value (NPV) of the quantitative electrocardiography (QCG) score and the consensus scores of the expert groups were calculated based on the Youden Index. The 95% confidence interval (CI) of the difference between the area under the curve (AUC) of the QCG score and the consensus score was calculated using DeLong’s method to confirm the non-inferiority of the biomarker over human experts’ consensus decision. Non-inferiority was confirmed if the lower margin of the 95% CI was larger than our predetermined non-inferiority margin (−0.05 difference). *p*-values < 0.05 were considered statistically significant. Data handling and statistical analyses were performed using the R-package version 3.3.2 (R Foundation for Statistical Computing, Vienna, Austria). 

## 3. Results

### 3.1. Baseline Characteristics 

A total of 82 OHCA patients were admitted to the emergency department and underwent CAG. A total of 75 of these 82 patients underwent a 12-lead ECG(s) within one hour of resuscitation. After the exclusion of a patient with VT ECG and 16 patients with vasospasm, a total of 58 patients were included in the study. Coronary occlusion (either near-total or total) was identified in 24 patients (Table 1). There were no significant differences in demographics, underlying diseases, 30-day survival, or discharge neurological outcomes. Emergency angiography was performed more frequently in patients with obstruction: 19 (79.2%) vs. 15 (44.1%).

**Figure 1:**
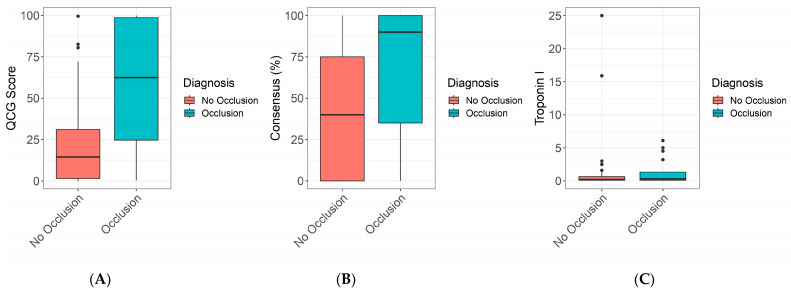
Boxplot of (**A**) QCG scores of the coronary occlusion group and the non-occlusion group. (**B**) Experts’ consensus of the two groups. (**C**) Troponin I level in the two groups.

Figure 1 and Table 1 show the differences in the QCG score, consensus score, and Troponin I between the two groups. The QCG score showed a significant difference, with the occlusion group showing a score of 0.625 (0.208–0.988) and the non-occlusion group showing a score of 0.145 (0.015–0.320) (*p* = 0.001). The Consensus Score also showed a significant difference: the occlusive group showed a score of 0.9 (0.3–1.0) and the non-occlusion group showed a score of 0.4 (0.0–0.8) (*p* = 0.020). Troponin levels showed no significant differences: 0.300 ng/mL (0.100–1.350) vs. 0.200 ng/mL (0.100–0.700) (*p* = 0.381).

### 3.2. QCG Biomarker Versus Expert Group

**Figure 2:**
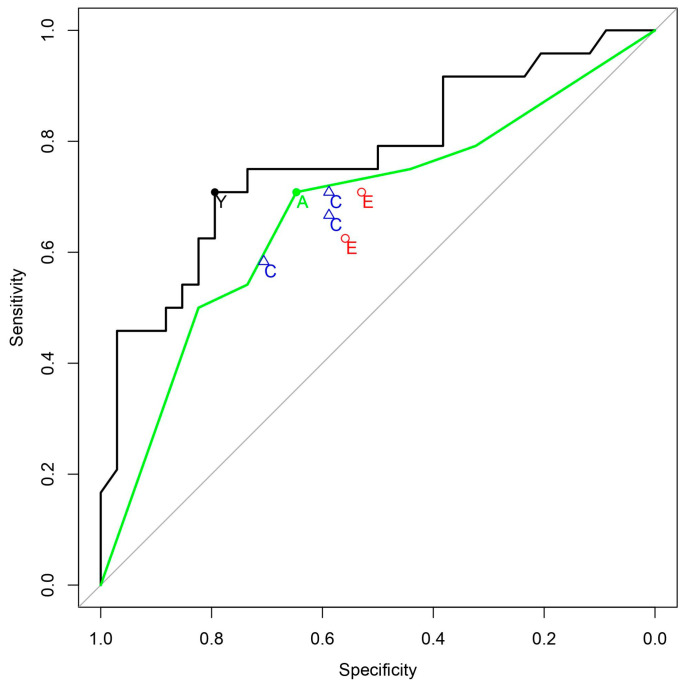
ROC curves for the prediction of coronary occlusion. Black solid line: ROC curve of QCG, Green solid line: ROC curve of experts’ consensus, Y (black filled circle): Youden Index Point of QCG, E (red hollow circles): Emergency Physicians, C (blue hollow triangles): Cardiologists, A (green filled circle): Youden Index Point of the Consensus score.

The classification ability of the QCG biomarker had an AUC of 0.770 (0.641–0.900). On applying a threshold of 0.376, the sensitivity was 70.8 (48.9–87.4), the specificity was 79.4 (62.1–91.3), the positive predictive value (PPV) was 70.8 (54.5–83.1), and the negative predictive value (NPV) was 79.4 (66.9–88.0) (Table 2, Figure 2). The classification abilities of emergency physicians and cardiologists varied from an AUC of 0.592 to 0.648. When combined into a consensus score, the expert group’s AUC was 0.676 (0.532–0.820), with a sensitivity of 70.8 (48.9–87.4), specificity of 64.7 (46.5–80.3), PPV of 58.6 (45.7–70.5), and NPV of 75.9 (61.6–86.0). 

The difference in classification ability between the QCG score and the consensus score was found to be 0.094 (−0.017–0.205). As the lower margin of this range was greater than −0.05, the QCG score was not inferior to the consensus of the five experts (Table 3).

## 4. Discussion

In this study, we tested the performance of an AI-based digital biomarker in screening for occlusive MI in out-of-hospital cardiac arrest patients. The biomarker demonstrated its non-inferiority compared to the combined judgment of cardiologists and emergency medicine physicians. Data in Table 3, showing the AUC confidence interval ranging from −0.017 to 0.205, suggests no significant difference, underlining our goal to prove the AI’s non-inferiority rather than its superiority. It is also worth considering that the AI was compared against the consensus score of five experts in cardiology and emergency medicine from a university hospital. Given that the sensitivity and specificity coordinates of the experts are all located below the ROC curve of the consensus score, we believe that our algorithm could be more useful in real clinical situations when evaluated by a single physician for making more accurate assessments. According to our previous study [15], in patients presenting with chest pain rather than cardiac arrest, the AUC for STEMI diagnosis was high in both AI and the consensus of experts, at 0.919 and 0.856, respectively. However, in the case of cardiac arrest in this study, the AUCs were relatively lower in both groups. This indicates that diagnosing coronary occlusion using the initial ECG of patients resuscitated after cardiac arrest is a challenging task. We believe this study is significant in demonstrating that the AI-based ECG biomarker achieved non-inferior results compared to a group of experts, even in cases of cardiac arrest. This research is the first of its kind globally, revealing the potential role of AI in decision-making regarding CAG in OHCA patients, especially in scenarios where specialized experts like cardiologists are not available.

The COACT trial, a significant study in the field of cardiac arrest management, explored the efficacy of immediate versus delayed coronary angiography in patients who had been resuscitated from out-of-hospital cardiac arrest without ST-segment elevation on ECG. The study included 538 participants, with a follow-up duration of 90 days and 1 year. It revealed that immediate angiography with the intent to revascularize was not superior to delayed angiography among these patients. The primary outcome measured was survival at 90 days, and secondary outcomes included survival with good cerebral performance, survival to hospital discharge, and incidences of major bleeding and need for renal replacement therapy [17,18]. The downsides of performing early CAG on patients following OHCA include logistical challenges and increased risk exposure. Early CAG necessitates moving patients at a time when they may be hemodynamically unstable, which can lead to exposure to contrast agents and entail procedural risks such as bleeding, stroke, and other complications [19]. Furthermore, prioritizing CAG as the primary intervention may not only delay other crucial, potentially life-saving treatments but also slow down the process of identifying the underlying cause, especially when a coronary lesion is not the trigger for the event. This approach underscores the need for careful consideration of the timing and prioritization of CAG in the acute management of OHCA patients [20]. This suggests that the benefit of CAG can be limited to the patient with actual acute coronary occlusion and therefore it is important to accurately identify such patients.

Cardiac troponins are the biomarkers of choice for acute myocardial infarction [21]. Troponin is released into the bloodstream 4–6 h after acute MI, and peaks after approximately 18–24 h [22]. According to Wereski R. et al., at presentation in STEMI patients, the median troponin concentration was 0.196 ng/mL, and in 2.2% of patients, it was less than 0.005 ng/mL. When a threshold of 0.052 ng/mL was applied, only 73.2% were reported as positive [23]. Therefore, the initial troponin level immediately after ROSC may not be diagnostic in deciding to proceed with emergency coronary angiography, highlighting the need for a more accurate and rapid assessment of the ECG.

In this context, the need for an objective and quantifiable ECG biomarker for acute coronary occlusion has become increasingly vital. AI’s ability to discern subtle patterns and variations in ECGs, which might be overlooked or misinterpreted in chaotic emergency settings, offers a significant advantage. By providing a more nuanced and detailed analysis of ECG data, AI can aid in more accurate identification of patients who may benefit from immediate CAG among resuscitated OHCA patients.

In this study, we used initial ECG only. However, ECG changes dynamically after ROSC, making it challenging to rely on a single ECG for clinical decision-making. This is corroborated by findings from the PEACE study, which indicate that early ECG acquisition post-ROSC is associated with a higher percentage of false-positive findings for STEMI [24]. In fact, ECGs obtained within 8 min post-ROSC showed a significantly higher false-positive rate compared to those obtained later. This suggests that delaying ECG acquisition by at least 8 min post-ROSC may improve its accuracy in diagnosing actual acute coronary occlusion, thus aiding in better patient selection for urgent CAG and minimizing false-positive diagnoses. Considering the dynamic nature of ECG changes post-ROSC, an approach that involves delayed or repeated ECGs could provide a more accurate and reliable assessment for guiding clinical interventions, such as CAG.

In this context, incorporating AI into serial ECG testing and harnessing its ability to provide a quantified digital biomarker for coronary occlusion may allow clinicians to objectively track the trend of coronary occlusion risk over time. As ECGs dynamically change post-ROSC, this approach may ensure that decisions regarding CAG are based on the trajectory of a measurable biomarker rather than a single snapshot in time. By observing changes in this biomarker, clinicians can make more accurate, objective, and reproducible decisions, potentially reducing unnecessary angiographies and focusing interventions on patients with a quantifiable high risk of occlusion.

Another promising aspect of the digital biomarker is its ability to utilize printed ECG images, enabling analysis through mobile phone cameras. This feature allows for the use of smartphone-based AI services even in chaotic environments of a resuscitation room. Traditional approaches, which rely on raw ECG data, often encounter practical challenges unless a data pipeline and AI integration are already in place. In contrast, our approach makes it exceptionally versatile and adaptable to various clinical settings, including those where traditional ECG data process infrastructures might be lacking.

This study has several limitations that warrant consideration. Firstly, its retrospective design may have introduced biases that could have affected the interpretation of results. Secondly, the study might have introduced selection bias by analyzing only patients who underwent CAG after ROSC. Thirdly, the number of patients analyzed in this study is less than 100, making it a relatively small-scale study. This was due to the low number of patients who underwent coronary angiography after cardiac arrest. Based on the results of this study, the authors plan to conduct further multi-institutional research based on the collection of data from multiple sources. Fourth, the study dataset was constrained in its ability to assess the diagnostic efficacy of the QCG for identifying new-onset left bundle branch block (LBBB), which is one of the diagnostic criteria for STEMI, because no cases of LBBB were found among those finally included in the study. Fifth, reliance on printed ECGs could raise concerns about image quality and interpretation. Finally, the AI algorithm’s diagnostic accuracy and clinical utility require further validation in larger, prospective studies to establish efficacy across diverse clinical settings.

## 5. Conclusions

An ECG-based digital biomarker could predict total or near-total coronary occlusion in resuscitated OHCA patients, and it was non-inferior to the consensus of a group of clinical experts. Combined with serial ECG analysis and other clinical information, it could provide objective and reproducible decision-making criteria for immediate angiography in resuscitated OHCA patients.

## Figures and Tables

**Table 1:** Patient Characteristics.

		Occlusion	No Occlusion	*p*
		(*n* = 24)	(*n* = 34)	
Age, mean (SD)		55.1 (10.4)	51.8 (13.5)	0.318
Sex, frequency (%)				1.000
	Male	21 (87.5)	29 (85.3)	
	Female	3 (12.5)	5 (14.7)	
Height, median (IQR)		170.0 (166.5–175.0)	170.0 (165.0–176.0)	0.623
Weight, mean (SD)		68.6 (10.1)	70.0 (12.9)	0.664
HTN, frequency (%)				1.000
	Yes	10 (41.7)	14 (41.2)	
	No	14 (58.3)	20 (58.8)	
DM, frequency (%)				0.883
	Yes	4 (16.7)	4 (11.8)	
	No	20 (83.3)	30 (88.2)	
CVD, frequency (%)				0.706
	Yes	5 (20.8)	8 (23.5)	
	No	17 (70.8)	21 (61.8)	
	Unknown	2 (8.3)	5 (14.7)	
Witnessed, frequency (%)				1.000
	Yes	20 (83.3)	29 (85.3)	
	No	4 (16.7)	5 (14.7)	
Bystander CPR, frequency (%)				0.474
	Yes	17 (70.8)	28 (82.4)	
	No	7 (29.2)	6 (17.6)	
30-day Survival, frequency (%)				0.755
	Yes	20 (83.3)	26 (76.5)	
	No	2 (8.3)	5 (14.7)	
	Unknown	2 (8.3)	3 (8.8)	
Discharge CPC, frequency (%)				0.765
	1	17 (70.8)	22 (64.7)	
	2	3 (12.5)	3 (8.8)	
	3	0 (0.0)	0 (0.0)	
	4	2 (8.3)	3 (8.8)	
	5	2 (8.3)	4 (11.8)	
	Unknown	0 (0.0)	2 (5.9)	
Emergency Angiography (<24 h), frequency (%)				0.016
	Emergency	19 (79.2)	15 (44.1)	
	Delayed	5 (20.8)	19 (55.9)	
Initial Rhythm (Prehospital), frequency (%)				0.583
	Shockable	21 (87.5)	29 (85.3)	
	Asystole	1 (4.2)	3 (8.8)	
	PEA	2 (8.3)	1 (2.9)	
	Unknown	0 (0.0)	1 (2.9)	
Initial Rhythm (ED Arrival), frequency (%)				0.172
	Shockable	5 (20.8)	1 (2.9)	
	Asystole	1 (4.2)	2 (5.9)	
	PEA	3 (12.5)	4 (11.8)	
	Prehospital ROSC	15 (62.5)	27 (79.4)	
Rhythm (After ROSC), frequency (%)				0.329
	Sinus Rhythm	8 (33.3)	15 (44.1)	
	Sinus Tachycardia	5 (20.8)	10 (29.4)	
	Atrial Fibrillation	7 (29.2)	4 (11.8)	
	Accelerated Junctional Rhythm	3 (12.5)	1 (2.9)	
	Atrial Flutter	0 (0.0)	1 (2.9)	
	Escape Bradycardia	1 (4.2)	0 (0.0)	
	Escape Capture Bigeminy	0 (0.0)	1 (2.9)	
	Supraventricular Tachycardia	0 (0.0)	1 (2.9)	
	Ventricular Bigeminy	0 (0.0)	1 (2.9)	
Troponin I *, ng/mL, median (IQR)		0.300 (0.100–1.350)	0.200 (0.100–0.700)	0.381
QCG, median (IQR)		0.625 (0.208–0.988)	0.145 (0.015–0.320)	0.001
Consensus Score, median (IQR)		0.9 (0.3–1.0)	0.4 (0.0–0.8)	0.2

* The reference value of troponin I: <0.028 ng/mL. SD: standard deviation, IQR: interquartile range, HTN: hypertension, DM: diabetes mellitus, CVD: cardiovascular disease, CPR: cardiopulmonary resuscitation, CPC: cerebral performance scale, PEA: pulseless electrical activity, ED: emergency department, ROSC: return of spontaneous circulation.

**Table 2:** Diagnostic accuracies of the QCG score and the experts.

	AUC (95% CI)	Threshold	Sensitivity (95% CI)	Specificity (95% CI)	PLR (95% CI)	NLR (95% CI)	PPV (95% CI)	NPV (95% CI)
QCG	0.770	0.376	70.8	79.4	3.44	0.37	70.8	79.4
	(0.641–0.900)		(48.9–87.4)	(62.1–91.3)	(1.69–6.99	(0.19–0.70	(54.5–83.1)	(66.9–88.0)
Consensus *	0.676	0.500	70.8	64.7	2.01	0.45	58.6	75.9
	(0.532–0.820)		(48.9–87.4)	(46.5–80.3)	(1.19–3.38)	(0.23–0.88)	(45.7–70.5)	(61.6–86.0)
EP#1	0.619	-	70.8	52.9	1.51	0.55	51.5	72.0
	(0.493–0.745)		(48.9–87.4)	(35.1–70.2)	(0.97–2.34)	(0.27–1.11)	(40.6–62.3)	(56.1–83.8)
EP#2	0.592	-	62.5	55.9	1.42	0.67	50.0	67.9
	(0.462–0.722)		(40.6–81.2)	(37.9–72.8)	(0.87–2.31)	(0.37–1.22)	(38.0–62.0)	(53.8–79.3)
CA#1	0.645	-	58.3	70.6	1.98	0.59	58.3	70.6
	(0.517–0.772)		(36.6–77.9)	(52.5–84.9)	(1.07–3.69)	(0.35–0.99)	(42.9–72.3)	(58.8–80.2)
CA#2	0.648	-	70.8	58.8	1.72	0.50	54.8	74.1
	(0.523–0.773)		(48.9–87.4)	(40.7–75.4)	(1.07–2.77)	(0.25–0.98)	(43.0–66.2)	(59.1–85.0)
CA#3	0.627	-	66.7	58.8	1.62	0.57	53.3	71.4
	(0.500–0.755)		(44.7–84.4)	(40.7–75.4)	(0.99–2.65)	(0.30–1.07)	(41.2–65.1)	(57.1–82.5)

* Consensus of experts. CI: confidence interval, PLR: positive likelihood ratio, NLR: negative likelihood ratio, PPV: positive predictive value, NPV: negative predictive value, EP: emergency physician, CA: cardiologist.

**Table 3:** Non-inferiority test.

Non-Inferiority Margin	Difference	95% CI	Non-Inferiority
−0.05	0.094	−0.017 to 0.205	Confirmed

CI: confidence interval.

## Data Availability

The original contributions presented in the study are included in the article. Further inquiries can be directed to the corresponding author/s.
